# Anaesthetic Management of Right Inguinal Hernioplasty With Hemiorchidectomy in a High-Risk Dilated Cardiomyopathy Patient

**DOI:** 10.7759/cureus.68871

**Published:** 2024-09-07

**Authors:** Prasanna Vadhanan, Karthik S Akella

**Affiliations:** 1 Department of Anaesthesiology, Vinayaka Mission's Medical College, Vinayaka Mission's Research Foundation, Karaikal, IND

**Keywords:** anaesthetic management, cardiac risk assessment, dilated cardiomyopathy, heart failure, hernioplasty, iliohypogastric block, ilioinguinal block, perioperative care, regional anesthesia, transversus abdominis plane block

## Abstract

This case report details the successful anaesthetic management of a 65-year-old male with severe dilated cardiomyopathy, coronary artery disease, and extensive cardiovascular risk factors scheduled for elective right inguinal hernioplasty with hemiorchidectomy. The anaesthetic approach, including regional nerve blocks (ilioinguinal, iliohypogastric, genital branch of genitofemoral) and transversus abdominis plane block, successfully minimised cardiac stress and maintained hemodynamic stability throughout the procedure.

## Introduction

Inguinal hernia repair is one of the most common surgical procedures performed worldwide, typically resulting in seven to 14 days of medical leave. Early complications may include bruising, numbness, and infection, while chronic pain is a notable long-term issue [1]. Patients generally seek minimal anaesthetic risk, reduced discomfort, swift recovery, and prompt discharge. Anaesthesia options for inguinal hernia repair include local infiltration, regional blocks, subarachnoid block, and general endotracheal anaesthesia. The choice of technique depends on factors such as the complexity and duration of the procedure, as well as the preferences of the surgeon and anaesthetist [2]. Day-case herniorrhaphy, whether under local anaesthesia (LA) or general anaesthesia (GA), proves to be more cost-effective and achieves comparable clinical outcomes to inpatient care, thus reducing financial burdens and inpatient bed demands without compromising patient care [3]. While both transversus abdominis plane (TAP) block and ilioinguinal-iliohypogastric (II/IH) block target the ilioinguinal and iliohypogastric nerves, TAP is a compartmental block and II/IH is a truncal block. TAP block offers comparable postoperative analgesia, but evidence increasingly supports that II/IH block provides superior pain control for inguinal hernia repair in both adults and children [4]. Additionally, the genitofemoral (GF) nerve, which innervates the inguinal region, may not be adequately addressed by these techniques alone. Therefore, adding a GF nerve block can enhance analgesia [5].

In patients with comorbidities, especially those with cardiac conditions such as dilated cardiomyopathy (DCM), careful planning of the anaesthesia strategy is crucial. DCM is associated with increased perioperative morbidity and mortality [6], and this risk is even higher in individuals with stage C heart failure [7]. Our patient, a 65-year-old male with a 20-day history of right inguinoscrotal swelling and recent onset of pain, was diagnosed with DCM and stage C heart failure. Following two weeks of intensive medical management, which significantly improved his cardiac tolerance, surgical intervention was undertaken due to persistent pain. Given his American Society of Anesthesiologists (ASA) physical status of 3, a combination of the ilioinguinal, iliohypogastric, and genital branch of the GF and TAP blocks was chosen to provide effective anaesthesia while minimizing systemic effects. This regional approach, supplemented with procedural sedation using fentanyl and a nitrous oxide and oxygen mixture, ensured hemodynamic stability throughout the procedure. This case highlights the importance of a careful, multimodal anaesthesia strategy in high-risk cardiac patients, demonstrating the benefits of combining regional techniques with balanced anaesthesia to optimise surgical outcomes.

## Case presentation

**Figure 1:**
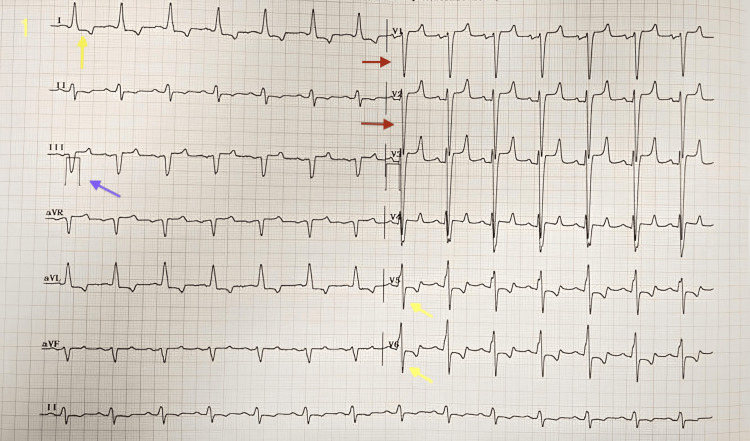
ECG. ECG findings reported the following: rate = 85/min, sinus rhythm, left axis deviation, QRS duration = 152 ms (>120 ms) (blue arrow), absent Q wave, broad R wave, discordant ST-T changes (ST depression and T wave inversion) in leads 1, v5, and v6 (yellow arrow), and deep S waves in v1 and v2 (red arrow).

**Figure 2:**
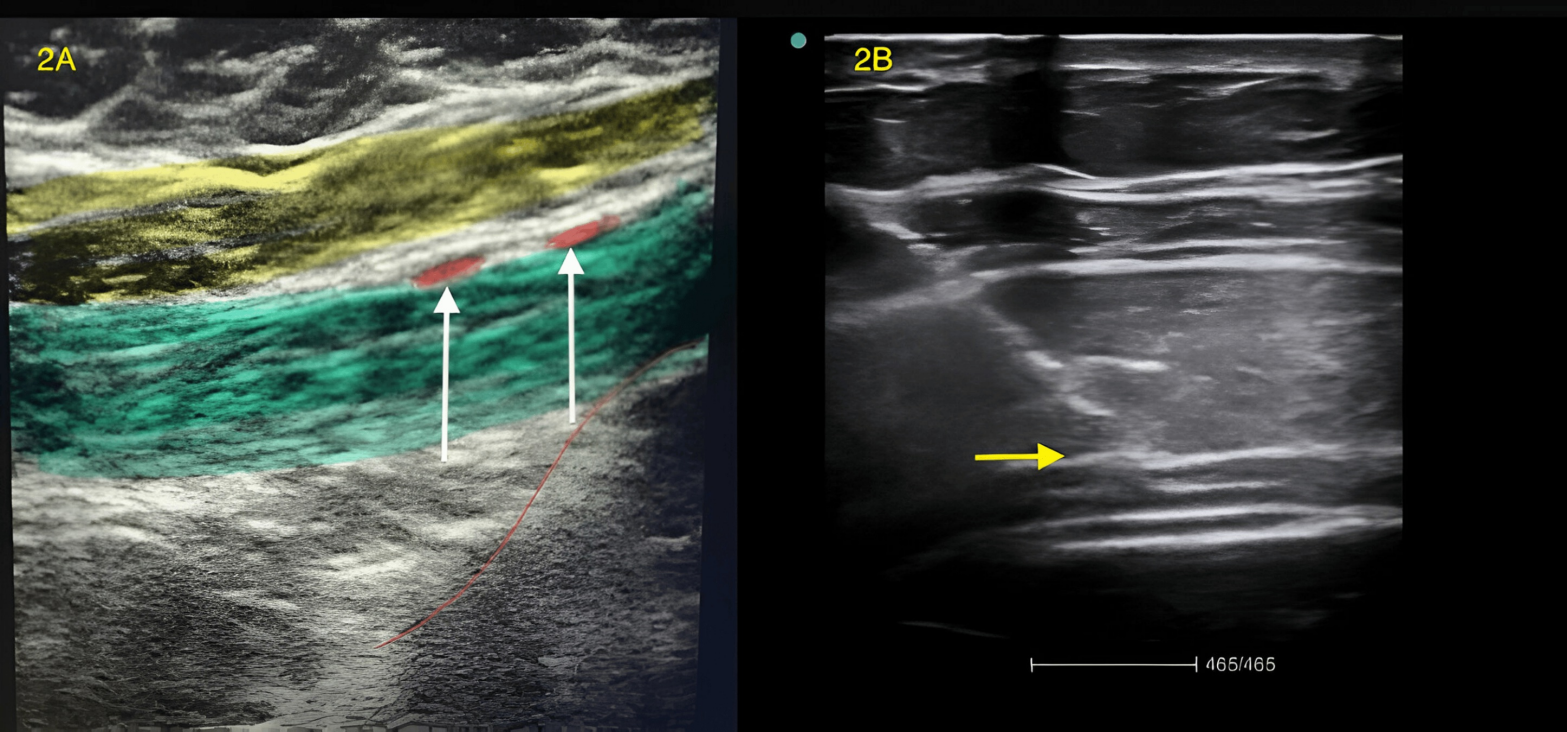
Ultrasound images showing Ilioinguinal, Iliohypogastric subcostal, and transversus abdominis plane block alone with intra-operative images. A: USG image of the ilioinguinal and iliohypogastric nerves (white arrows). B: Transversus abdominis plane block and the subcostal nerve (yellow arrow).

A 65-year-old, 80 kg male presented with right inguinoscrotal swelling persisting for 20 days with a recent onset of pain. He was wheelchair-bound due to breathlessness (New York Heart Association class 3) and had a history of orthopnoea. On examination, his pulse rate was 90/minute and his blood pressure was 100/60 mmHg. He had bilateral basal crepitations and an S3 was auscultated. His haemoglobin was 8.9 g%, chest X-ray showed cardiomegaly, and ECG (Figure 1) findings suggested left bundle branch block. A cardiologist's opinion was obtained, and echocardiography findings indicate an ejection fraction (EF) of 16%, global hypokinesia of the left ventricle, moderate mitral and tricuspid regurgitation, moderate pulmonary artery hypertension, and grade 3 diastolic dysfunction. The left ventricular end-diastolic diameter (LVEDD) was 71 mm, with an interventricular septum (IVS) thickness of 10 mm and posterior wall thickness of 10 mm. The patient, who was 6 feet tall, weighed 80 kg, and had a body surface area of 2 m², had a left ventricular mass index (LVMI) of 163.59 g/m², which is considered severely abnormal in males (values above 149 g/m²). Additional findings included global hypokinesia, an E-wave velocity of 120 ms (<160 ms indicates a restrictive filling pattern), an E/A ratio greater than 2 (suggesting a restrictive pattern), and an elevated E/E' ratio. With an EF of just 16%, these results were indicative of dilated cardiomyopathy (DCM) [8]. The Revised Lee’s Cardiac Risk Assessment Index classified the patient as having a class 3 risk (10.1%). Consequently, the patient was diagnosed with DCM and stage C heart failure with reduced ejection fraction (HFrEF). He was started on tablets atorvastatin 10 mg once a day (od), clopidogrel 75 mg od, torsemide 10 mg od, metolazone 2.5 mg od, nicorandil 10 mg od, ivabradine 5 mg twice a day (bd), and taurine (500 mg) + acetylcysteine (150 mg) bd, along with iron, folic acid, and calcium supplementations. After two weeks of treatment, the patient exhibited significant improvement in functional capacity, being able to walk a few feet without dyspnoea and lie down on flat surfaces. The patient developed increasing pain and tenderness necessitating immediate surgery (hernioplasty). The patient was assessed under ASA physical status 3. The plan of anaesthesia was combined ilioinguinal (II), iliohypogastric (IHN), genital branch of genitofemoral (GFN), and transversus abdominis plane (TAP) blocks (Figure 2). All medications except torsemide were continued, and the patient fasted for solids for four hours.

**Figure 3:**
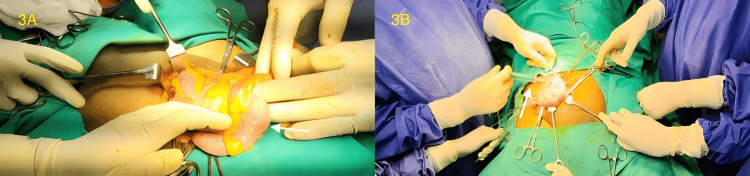
Intraoperative images. A: Contents of the hernia (white arrow). B: Surgeon infiltrating the hernial sac (white arrow).

In the operating theatre, an 18G IV cannula was secured. Standard monitors were attached (non-invasive blood pressure (NIBP), 5-lead ECG, and oxygen saturation (SpO2)). The patient received procedural sedation with fentanyl 20 mcg injection along with Ringer's lactate at 10 ml/kg/hour as maintenance. A block mixture of 15cc of 2% lignocaine with adrenaline and 15cc of 0.5% bupivacaine with 15cc of sterile water was prepared. Under sterile precautions, a linear high-frequency probe (Sonosite M-Turbo, FUJIFILM Sonosite, Inc., Bothell, WA) was used and ultrasound-guided right ILN (10cc), right IHN (10cc), and the genital branch of GFN (10cc), along with posterior TAP block (15cc) (Figure 2) were administered. An infusion of dexmedetomidine 50 mcg bolus over 15 minutes followed by an infusion of 25 mcg/hour was administered. Additionally, surgeons infiltrated the site of the incision and the hernial sac (Figure 3) with bupivacaine 0.25% (10cc). On the table, the surgeon decided to proceed with right hemiorchidectomy, necessitating an additional 20 mcg of fentanyl with inhalation of 50:50 nitrous oxide and oxygen mixture via Bain's circuit for a total duration of 15 minutes. Haemodynamics remained stable throughout the procedure, and the heart rate and mean arterial pressures were within 10% of the baseline values. Postoperatively, the patient was responsive and reported adequate pain relief (visual analogue scale (VAS) 3) for up to 13 hours, after which mild discomfort (VAS 5) was noted, which was treated with paracetamol 1 g intravenous infusion and ketorolac 30 mg intramuscularly. Postoperative 12-lead ECG showed no new changes. He was mobilised on the first postoperative day and discharged after three days. On follow-up after a week, he was comfortable and showed renewed capabilities while performing daily activities.

## Discussion

Anaesthetic management of patients with heart failure with reduced EF is challenging in low-resource settings. The reported incidence of major adverse cardiac events (MACE) in such patients is 15.2% within 30 days [9]. Central neuraxial blocks might cause refractory hypotension, which might worsen myocardial ischemia [10]. Ultrasound-guided IL/IHN is a widely validated anaesthetic technique for inguinal hernias and the addition of GFN is associated with lower postoperative VAS scores and lower doses of intraoperative skin and sac infiltrations [11]. The TAP block was administered to provide adequate muscle relaxation for reducing the large hernia. The subcostal nerve is also reliably blocked by this technique [12]. The mesenteric pull did cause some discomfort to the patient; fentanyl boluses effectively alleviated it. The surgeon's decision to perform an orchidectomy greatly reduced the procedural timings, aiding in early recovery. The unexpected intraoperative decision to proceed with a hemiorchidectomy was managed with a 50:50 inhalation mixture of nitrous oxide and oxygen. While inhalation anaesthetics can produce a dose-dependent reduction in mean arterial pressure (MAP), nitrous oxide is the exception [13]. Concerns about nitrous and pulmonary hypertension exist; however, the increase can be modest and not associated with significant changes in other parameters [14]. The duration of surgery was also brief, which plays an important role in haemodynamic stability and postoperative recuperation. Postoperatively, excellent analgesia was maintained with a constant VAS score of less than 3 for 13 hours.

## Conclusions

In conclusion, a judicious combination of regional techniques and balanced anaesthesia using intravenous and inhalation anaesthesia resulted in the successful anaesthesia management of the patient with HFrEF without adverse events. The addition of TAP to the established GFN and II/IHN blocks is unique as it is associated with lower overall postoperative VAS scores, lower doses of intraoperative local anaesthetics, and lower procedural sedation doses. Furthermore, the addition of TAP even adds to this analgesic effect, furthering the aim of lower doses of local anaesthetics and analgesics for better analgesia over the postoperative days.
